# One Step Away From Extinction: A Population Genomic Analysis of A Narrow Endemic, Tropical Plant Species

**DOI:** 10.3389/fpls.2021.730258

**Published:** 2021-09-23

**Authors:** Thais M. Teixeira, Alison G. Nazareno

**Affiliations:** ^1^ Department of Genetics, Ecology and Evolution, Federal University of Minas Gerais, Belo Horizonte, Brazil; ^2^ Department of Ecology and Evolutionary Biology, University of Michigan, Ann Arbor, MI, United States

**Keywords:** demographic history, Fabaceae, *Mimosa catharinensis*, conservation genetics, genetic diversity

## Abstract

Intraspecific genetic variation plays a fundamental role in maintaining the evolutionary potential of wild populations. Hence, the assessment of genetic diversity patterns becomes essential to guide biodiversity conservation policies, particularly for threatened species. To inform management strategies for conservation of *Mimosa catharinensis* – a narrow endemic, critically endangered plant species – we identified 1,497 unlinked SNP markers derived from a reduced representation sequencing method (i.e., double digest restriction site associated DNA sequencing, or ddRADseq). This set of molecular markers was employed to assess intrapopulation genetic parameters and the demographic history of one extremely small population of *M. catharinensis* (*N*=33) located in the Brazilian Atlantic Forest. Contrary to what is expected for narrow endemic and threatened species with small population sizes, we observed a moderate level of genetic diversity for *M. catharinensis* [*uH*_E(0%missing data)_=0.205, 95% CI (0.160, 0.250); *uH*_E(30%missing data)_=0.233, 95% CI (0.174, 0.292)]. Interestingly, *M. catharinensis*, which is a lianescent shrub with no indication of seed production for at least two decades, presented high levels of outcrossing [*t*_(0%missing data)_=0.883, SE±0.0483; *t*_(30%missing data)_=0.909, SE±0.011] and an apparent absence of inbreeding [*F*_(0%missing data)_=−0.145, 95% CI (−0.189, −0.101); *F*_(30%missing data)_=−0.105, 95% CI (−0.199, −0.011)]. However, the reconstruction of demographic history of *M. catharinensis* indicated that the population should be suffered a recent bottleneck. Our population genomic study tackles a central issue in evolution and conservation biology and we expect that it will be useful to help safeguard the remaining genetic diversity reported for this unique genetic resource.

## Introduction

Differences in DNA sequences among individuals within a population represent its genetic diversity, a key component for the long-term survival of natural populations that is related to the maintenance of its evolutionary potential in the face of environmental change (e.g., Huenneke, 1991; Reed and Frankham, 2003; Spielman et al., 2004; Elegren and Galtier, 2016; Frankham et al., 2017; but see Zimmermann et al., 2018). As a matter of fact, the impact of the loss of genetic variation on increasing risk of extinction for wild populations has been extensively debated in the past (e.g., Lande, 1988; Caro and Laurenson, 1994; Frankham, 1995; Hedrick et al., 1995; Caughley and Gunn, 1996; Frankham and Ralls, 1998; Dobson, 1999), suggesting that demographic parameters (e.g., population size, density, sex ratio, age structure, fecundity; see Tarsi and Tuff, 2012) associated with stochastic and deterministic events should affect species on the verge of extinction before microevolutionary forces take effect (e.g., Frankham et al., 2010). Nonetheless, Spielman et al. (2004) reported that most threatened taxa, including plant species, present lower levels of genetic diversity than closely related non-threatened taxa, implying a higher risk of (local) extinction due to small population sizes, and as a consequence, a reduction in reproductive fitness.

Although the amount of genetic diversity in a population is commonly linked to its size and range (e.g., Frankham, 1996; Hamrick and Godt, 1996; Elegren and Galtier, 2016), a better understanding is needed of the susceptibility of species that are rare, endemic, and with small population sizes to extinction due to reductions in genetic diversity. Species that present small population sizes and a restricted geographic range (i.e., narrow endemic) tend to present lower levels of genetic diversity than those with larger population sizes and wide distribution (Kimura and Crow, 1964; Hamrick and Godt, 1989; Honnay and Jacquemyn, 2007; but see Ellis et al., 2006; Turchetto et al., 2016; Forrest et al., 2017; Martel et al., 2021). For instance, Honnay and Jacquemyn (2007) highlight significantly lower levels of genetic variation in small plant populations compared to large populations. The same pattern (i.e., low levels of genetic diversity) has been reported for threatened plant species with a history of fragmentation and/or population decline (e.g., Jiménez et al., 2014; Bupp et al., 2017; Simmons et al., 2018; Downey and Baskauf, 2020). In addition, high levels of inbreeding are expected for plant species with small and isolated populations (e.g., Falconer, 1989; Barret and Kohn, 1991; Allendorf and Luikart, 2007; Frankham et al., 2014; Oleas et al., 2014; Perrier et al., 2017; Rhoads et al., 2017; Toczydlowski and Waller, 2019; but see Nazareno and Carvalho, 2009; Guidugli et al., 2016), making the effects of genetic drift more pronounced. As a result, such populations are particularly prone to (local) extinction, as they lose variability more readily when compared to populations in which drift is an unexpressive microevolutionary force (e.g., Barret and Kohn, 1991; Bani et al., 2018; Toczydlowski and Waller, 2019). In addition to genetic factors, small populations are subject to rapid decline and extinction due to demographic fluctuations and environmental changes (e.g., Primack and Rodrigues, 2001; Frankham et al., 2010).

In this study, we aimed to assess the levels of genetic diversity and demographic history of a unique population of *Mimosa catharinensis* Burkart (Fabaceae), a rare, critically endangered (CONSEMA, 2014), and narrow endemic species that occurs in a small area (~700m^2^) in the Brazilian Atlantic Forest (Burkart, 1979). Considering the vulnerability of *M. catharinensis* to extinction and its unique reproductive biology (i.e., a plant species that is ecologically sterile), and based on the assumptions of population theory and the findings of empirical studies about genetic diversity in rare and endemic plant species occurring in small populations, we expected to find low levels of genetic diversity for *M. catharinensis*. Further, we expected to find indications of population retraction given the historic threats that have occurred in its biome (presented in the below section). To this end, and to contribute to *in situ* and *ex situ* conservation strategies, we used a high-throughput sequencing approach (i.e., double digest restriction site associated DNA sequencing, ddRADseq; Peterson et al., 2012) to identify unlinked and neutral SNP markers. This reduced representation sequencing method had been used extensively for species conservation studies (e.g., Nazareno et al., 2017; Chattopadhyay et al., 2019; Amor et al., 2020; Wright et al., 2020; Bard et al., 2021; Nazareno and Knowles, 2021) mainly due to its robustness to generate thousands of neutral and non-neutral molecular markers at a relative low cost. Our population genomic study tackles a central issue in evolution and conservation biology and we expect that it will be useful to safeguard the genetic diversity reported for the only remaining population of *M. catharinensis*. The approach applied in this study can also be used to guide conservation efforts for other plant species on the brink of extinction.

## Materials and Methods

### Species Description

**Figure 1:**
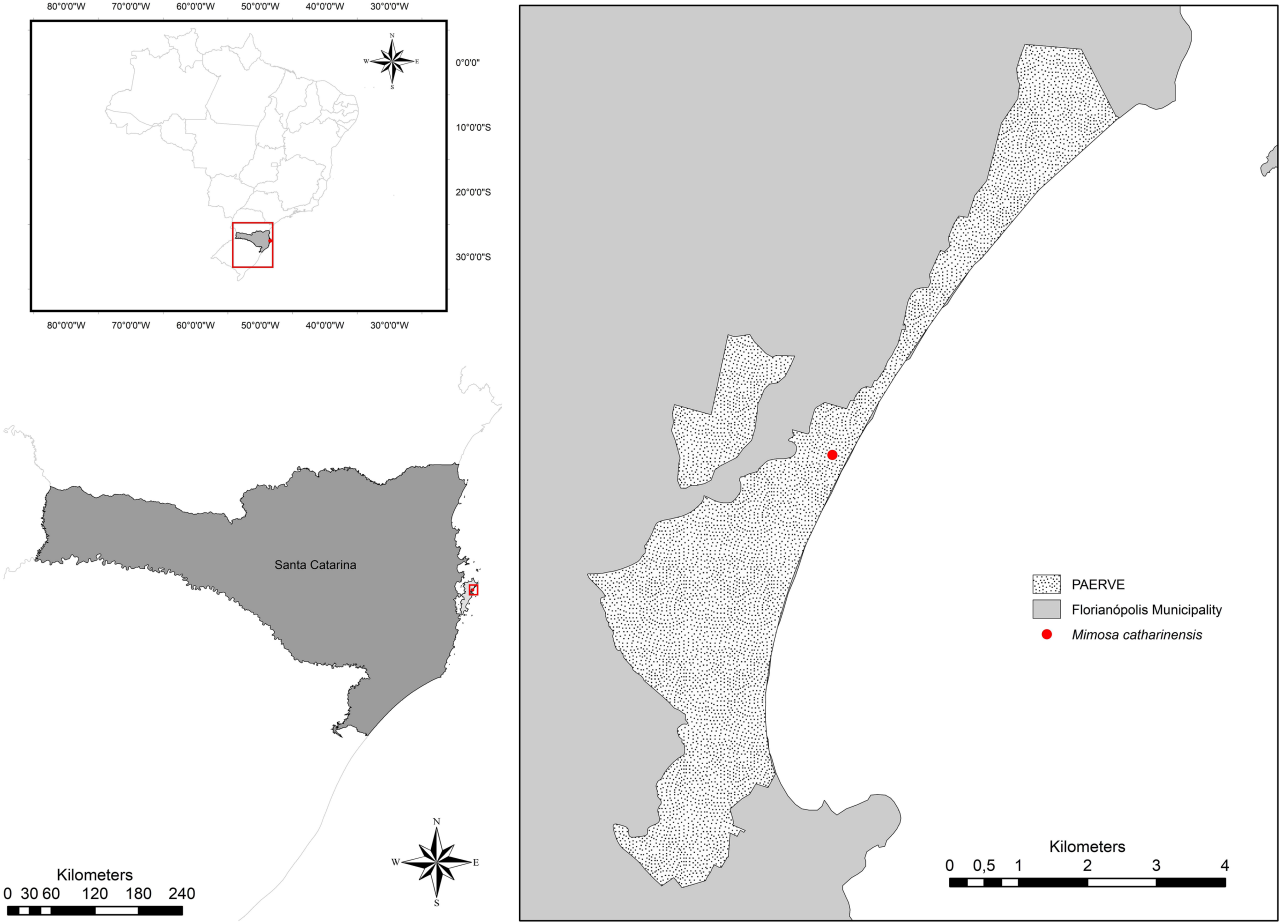
Location of the extremely small and unique population of *Mimosa catharinensis* in Rio Vermelho State Park (*Parque Estadual do Rio Vermelho*; PAERVE), Santa Catarina State, Southern Brazil.

*Mimosa catharinensis* is a plant species endemic to the Brazilian Atlantic Rainforest with an extremely restricted distribution, limited to one population located in the Rio Vermelho State Park (*Parque Estadual do Rio Vermelho* – PAERVE; Figure 1), Santa Catarina State, Southern Brazil (Burkart, 1979; Ferreira, 2010). The only documented population of *M. catharinensis* (~700m^2^) occurs in one of the most heavily impacted areas of the PAERVE (Figure 1) due to deforestation and environmental degradation for over 200years (Berenhauser, n.d.). This narrow endemic and critically endangered plant species (CONSEMA, 2014) is a lianescent shrub that produces masculine and hermaphrodite white, glabrous, and tetramerous flowers (Burkart, 1979). Pollen dispersal occurs *via* zoophily (e.g., *Apis mellifera*; Silva et al., 2005), although nectar-dependent visitors are infrequent due to a lack of floral nectar (Silva et al., 2005). The pods are of the indehiscent craspedium type and are linear-oblong with curved prickles on the margins (Medeiros and Stefani, 2018). Although the reproductive structures of *M. catharinensis* have been characterized morphologically, pods without seeds were observed during field collection in 2019. Based on herbarium records, the lack of seed production in *M. catharinensis* was first recorded in 1994 (Voucher 30,482; FLOR herbarium, UFSC). As no seedlings were found in its area of occurrence, *M. catharinensis* may be considered an ecologically sterile plant species (i.e., a plant species in which the recruitment rate over time is nil as a consequence of lack of sexual reproduction). As successful *in vitro* pollen germination had been reported for *M. catharinensis* (Silva et al., 2005), further pollination studies are needed to improve our understanding of its reproductive biology.

Although there are efforts by the Santa Catarina State government (Gasper et al., 2012) to generate genetic and ecological data to inform conservation efforts and to safeguard plant species threatened with extinction, no previous evolutionary studies – including phylogenetic analysis and genomic characterization – have been conducted on this critically endangered species.

### History of the Research Area

Since 2002, the PAERVE has been recognized by UNESCO as one of the core areas of the Atlantic Forest Biosphere Reserve and consists of 1,530 hectares of Atlantic Rainforest biome (Ferreira, 2010; Bechara et al., 2013). The introduction of pine and eucalyptus into the park has brought about significant changes to its *restinga* (sandy plains) vegetation and is the main cause of degradation of the coastal ecosystem of Santa Catarina Island (Ferreira, 2010). Currently, it is estimated that 750ha of the park are covered by *Pinus* spp., of which about 33% are located in dunes and sandbanks that have been progressively invaded since pine was first introduced (Bechara et al., 2013). Nevertheless, approximately 400ha of dense rainforest and 250ha of *restinga* vegetation fragments remain protected in the park (Bechara et al., 2013), the latter located mainly at the southern and northern tips of the conservation area (Ferreira, 2010).

### Population Sampling

We collected leaf samples from all 33 identified adult individuals of unknown age that constitute the only remaining *M. catharinensis* population. Leaves were stored in silica gel for later extraction. One voucher (FLOR30482) is deposited at the FLOR herbarium at the Federal University of Santa Catarina – UFSC.

### Genomic Library Preparation and Sequencing

DNA was extracted from leaf samples of all collected individuals employing the NucleoSpin® kit (Machereney-Nagel GmbH & Co. KG) following manufacture’s guidelines. After extraction, the quality of each sample was verified using Thermo Scientific NanoDrop 2000 Spectrophotometer (Thermo Fisher Scientific Inc.) and the concentration of double-strand DNA (dsDNA) was obtained by using Qubit dsDNA Assay Kit (Invitrogen). The genomic library was prepared using a double-digest restriction site-associated DNA sequencing (i.e., ddRADseq) protocol (Peterson et al., 2012) with modifications proposed by Nazareno et al. (2017). Digestions reactions were performed using 0.5μg of genomic DNA, 5,000 units of *MseI*, 5,000 units of HF-*EcoRI*, and 1x CutSmart buffer (New England Biolabs) in a total 20μl reaction volume for 3h at 37°C, ending with a 20min deactivation step at 65°C (Nazareno et al., 2017). Reactions products were cleaned using Agencourt AMPure XP system (Beckman Coulter) according to manufacturer’s recommendations. The dsDNA was quantified using Qubit and the amount of DNA was standardized before to procedure with the ligation. We used 80ng DNA, 1M of *MseI* adaptor, 0.33M of *EcoRI* double-strand adaptor unique for each sample, 1U of T4 DNA ligase (New England Biolabs) and 1.40μl of T4 ligase buffer in 30μl ligation reaction that was stored at a room temperature (23°C) for 30min After, heat-killed the reaction at 65°C for 10min and then, slowly cooled until reach room temperature. Details of barcode sequences can be found in Nazareno et al. (2017). Products of the ligation reaction were purified using the Agencourt AMPure XP system and used in PCR reactions. PCR reactions were carried out in a total volume of 20μl, containing 13.5μl of ligation product, 0.2M dNTPs, 1.0μM MgCl_2_ 0.5U iProof™ High-Fidelity DNA Polymerase (Bio-Rad) and 2X of iProof buffer. The PCR protocol (98°C for 30s, 20cycles of 98°C for 20s, 60°C for 30s and 72°C for 40s, followed by a final extension at 72°C for 10min) was performed in an Eppendorf System. The amplicons were cleaned with the Agencourt AMPure XP system and quantified using Qubit dsDNA assay Kit. We used an automated size-selection technology (i.e., Pippin Prep; Sage Science, Beverly, MA, United States) at 2% agarose cartridge to select DNA fragments at a target range size of 375–475bp. The library was sequenced (100-bp single-end) on a lane of Illumina HiSeq 2,500 flow cell (Illumina Inc., San Diego, CA, United States) at The Centre for Applied Genomics in Toronto, Canada.

### Raw Data Processing and SNPs Identification

Data quality was checked using the program FastQC version: 0.11.8.^1^ The file containing raw sequence reads was analyzed in Stacks 2.41 (Catchen et al., 2011, 2013; Rochette et al., 2019) using *de novo* assembly. Initially, we used the process_radtags program in Stacks to examine individual barcodes, enzyme cutsite integrity and to demultiplex the data. For barcodes rescue we admitted at most two mismatches (−barcode_dist 1 2). We filtered raw reads using the phred score 33 and used the option −t to trim all of it at 85 base pairs (−phred 33, −t85). With the process_radtags output, we run the Ustacks program, that uses a maximum likelihood framework to aligns short-read sequences and create putative alleles (stacks). The maximum distance allowed between “stacks” was two nucleotides. The minimum depth of coverage required to create a “stack” and the maximum number of stacks at a single *de novo* locus was set as three. We enabled the deleveraging algorithm (−d) to solve merged tags, and a bounded-error model to identify SNPs. An alpha value of 0.05 and an upper bound of 0.1 were used. In the next step, we performed Cstacks to merge alleles of all samples and create a catalog of consensus loci. Three mismatches were allowed between loci in catalog build. Each individual sample loci were then compared against the catalog through the Sstacks program. We ran tsv2bam program to organize the single-end reads by locus, instead by sample, creating a BAM file that was used as input to the Gstacks program. Gstacks uses the single-reads to build contigs and merges them into loci. It also aligns reads to the loci using Ukkonen’s algorithm, identifies SNPs for each locus and each individual genotype and converts SNPs into phased haplotypes. Finally, we used POPULATIONS in Stacks (Catchen et al., 2011, 2013; Rochette et al., 2019) to generate and export the SNP data set, as well as FASTA files containing the per-locus consensus sequences, and individual loci sequences, that were applied in the further analyses. To assess the effects of missing data on genetic estimates, we run POPULATIONS several times with a Minor Allele Frequency (MAF) of 5% (−min_maf 0.05), a maximum heterozygosity (−max_obs_het) of 0.65, and the percentage of missing data varying from 0 to 30%. All data sets include one random SNP per locus. To avoid potential bias sources in the forward genetic analyses, Hardy–Weinberg (H–W) equilibrium tests was done using the adegenet and pegas packages^2^ (Jombart, 2008; Paradis, 2010; Jombart and Ahmed, 2011) implemented in R. In addition, linkage disequilibrium (LD) between loci was tested using Arlequin 3.5.2 (Excoffier and Lischer, 2010). Type I error rates for these tests were corrected for multiple comparisons using the sequential Bonferroni procedure (Rice, 1989), and SNPs that failed the H–W equilibrium test and/or SNP pairs in LD were excluded from further analyses. To exclude non-nuclear loci, the per-locus consensus sequences were aligned against reference chloroplast and mitochondrial genomes using *Acacia dealbata* (NCBI accession number KX852435) and *Acacia ligulata* (NCBI accession number MH933866), respectively. We used the *BLASTn* program^3^ to identify loci that presented identity greater than or equal to 80%.

### Genetic Diversity

To assess the influence of missing data on genetic diversity estimates, we used the “BasicStats” function in the DiveRsity package in the R software environment^4^ (Keenan et al., 2013). We estimated unbiased expected genetic diversity (*uH*_E_; Nei and Roychoudhury, 1974), observed heterozygosity (*H*_O_), and the inbreeding coefficient (Wright’s Fixation Index *F*_IS_; Wright, 1943). Population genetics statistics were averaged across loci using the DiveRsity package in R (Keenan et al., 2013). Confidence intervals at 95% were obtained to evaluate differences among means of genetic parameters estimated for all data sets. We used the final data set to calculate minor allele frequencies using the adegenet package in R (Jombart, 2008; Jombart and Ahmed, 2011).

### Mating System of *Mimosa catharinensis*

We used the kinship coefficient (Loiselle et al., 1995) implemented in the SPAGeDi program (version 1.5; Hardy and Vekemans, 2002) to estimate random outcrossing rates (1-*s*, where *s* indicates the estimated selfing rate) based on standardized identity disequilibrium for all data sets. Significance for the identity disequilibrium coefficient was obtained with 1,000 permutations, and a jackknife over loci approach was applied to calculate the standard error of outcrossing estimates.

### Clonality Assessment

We evaluated the power of discrimination (PD) for the complete single nucleotide polymorphism (SNP) data set (i.e., the data set without missing data; MD) using the equation 1-PI, where PI represents the probability that two individuals drawn at random from a population will have the same genotype at multiple loci (Waits et al., 2001). This analysis was performed in GeneAlEx 6.5 program (Peakall and Smouse, 2012). Taking into account that clonal reproduction has been reported for plant species of the Fabaceae family (e.g., Dev et al., 2010; Gui et al., 2013; Gaddis et al., 2014; Mori et al., 2018; Amor et al., 2020), including *M. catharinensis* (Rogalski et al., 2005), a clonality test was applied for *M. catharinensis* using the “poppr” package (Kamvar et al., 2014, 2015) in R version 3.5.3 (R Core Team, 2019). The “bitwise.dist” function was used to calculate a pairwise genetic distance matrix between individuals. Then, we ran the “mlg.filter” function to apply a threshold that defines the minimum distance to determine distinct multi-locus genotypes (MLGs; see Kamvar et al., 2015). Applying a threshold when using SNP markers is important in order to deal with the limited observable genetic differences between genets due to sporadic somatic mutations or issues associated with high-throughput sequencing technologies (e.g., genotyping errors and missing data; Kamvar et al., 2015; Mastretta-Yanes et al., 2015). We chose the average neighbor as the clustering algorithm with a threshold of 0.04, which has been shown to be sufficient to explain intra-clonal variation when SNPs derived from ddRADseq are used (Amor et al., 2020). In addition, a less conservative threshold of 0.06 was used to infer the numbers of MLGs in *M. catharinensis*. The threshold of 0.06 was chosen in order to minimize the limitation of our experimental design, which did not include replicates to estimate genotyping errors. To visualize the number of putative lineages, we ran the “upgma” function (Phangorn package; Schliep, 2011) to construct ultrametric trees.

### Demographic History

**Figure 2:**
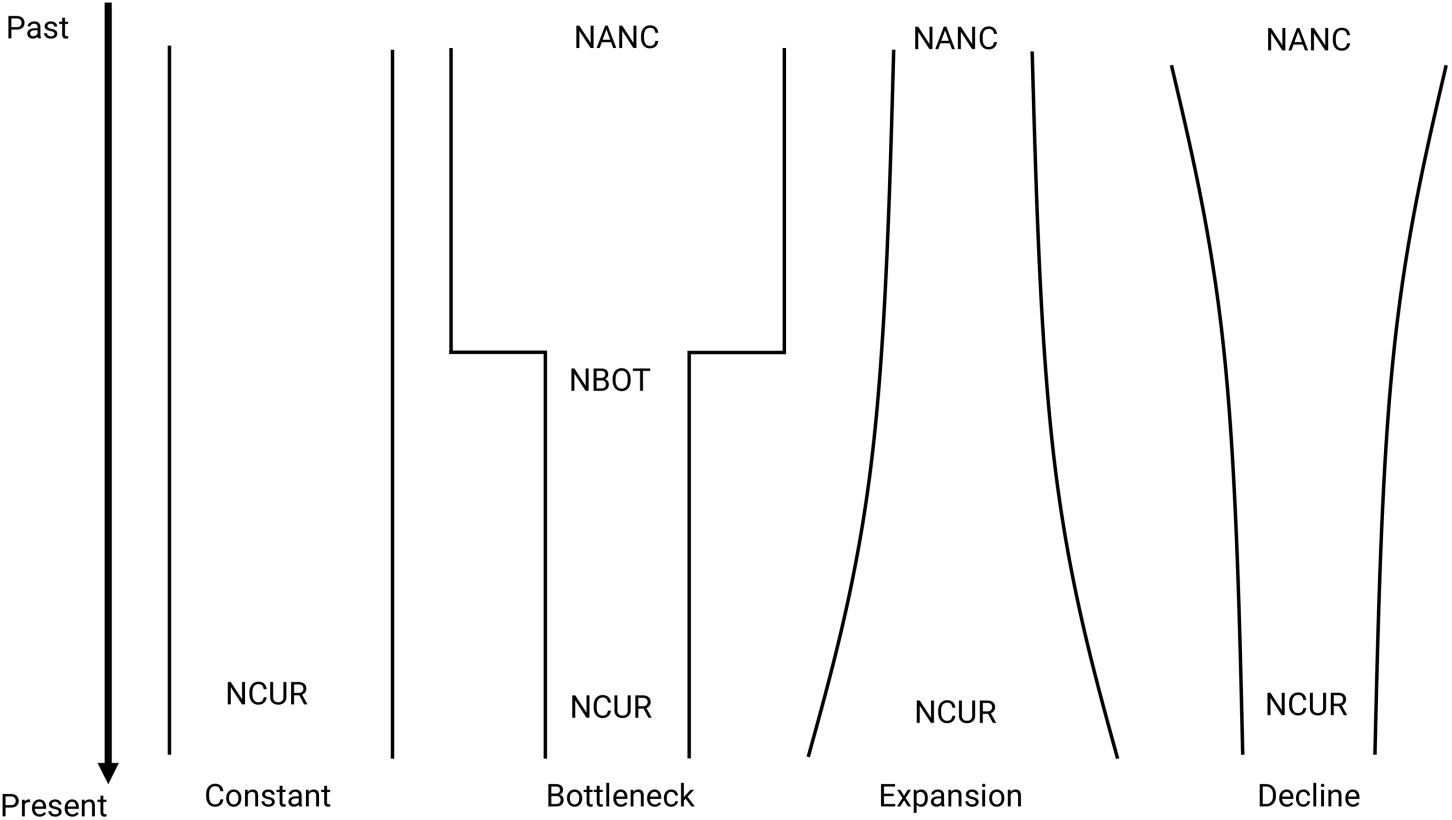
Representation of the demographic scenarios tested for *M. catharinensis* in FastSimCoal 2.6. Estimates include coalescent-based current (NCUR) and ancestral (NANC) population size, number of generations since the bottleneck occurred (TBOT), and population size at the end of the bottleneck (NBOT).

The demographic history of *M. catharinensis* was inferred using a composite likelihood-based approach implemented in FastSimCoal v. 2.6 (Excoffier et al., 2013). VCF files were converted into the *.arp format using the program PGDSpider 2.1.1.5 (Lischer and Excoffier, 2012). Assuming genetic distances of 0.04 and 0.06, we obtained two observed folded Site Frequency Spectrums (SFS based on minor allele frequency) for our filtered SNP data set using the Arlequin 3.5 program (Excoffier and Lischer, 2010). We tested four evolutionary scenarios: constant population size, bottleneck, population decline, and population expansion (Figure 2). We performed 100 replicates for each tested model and folded SFS. The parameters for each run were estimated based on 100,000 simulations and 40 ECM optimization cycles. We used an overall substitution rate of 7×10^−9^ per site/generation as reported for *Arabdopsis thaliana* (Ossowski et al., 2010). The maximum estimated likelihood for each demographic scenario and SFS were used to identify the model with the best fit, which was chosen based on Akaike’s Information Criterion (AIC; Akaike, 1973, 1974) estimated as ΔAIC. All models were ranked, with the model with the lowest ΔAIC being considered the most plausible. It is important to note that models with ΔAIC ≤2 have substantial support (Burnham and Anderson, 2001). We also assessed the probability that each model is the best fit by estimating Akaike weights (*w*_i_). Once the best model was selected, its estimated parameters were used to simulate 100 SFS data sets to built confidence intervals based on bootstrap distribution.

## Results

### Raw Data Processing and SNP Calling

The Illumina sequencing generated a total of 79,759,227 raw reads, of which less than 1% was discarded due to low quality. The average (±SE), minimum, and maximum retained reads were 2,384,161 (±115,118 SE), 1,470,849, and 4,011,918. The total number of genotyped RADtag loci was 274,113 with a mean coverage depth of 12.7 (±2.7 SD). After Bonferroni adjustment, significant deviations from Hardy–Weinberg equilibrium were observed for all data sets (Table 1). No linkage disequilibrium was observed between loci after Bonferroni correction for k tests (Table 1). After alignment, one locus matched the plastome of *A. dealbata* (96.47% identity) and it was excluded from the data set. The final numbers of unlinked SNPs used in each data set are shown in Table 1.

**Table 1:** Variation of genetic parameters (*H*_O_, observed heterozygosity; *uH*_E_, unbiased expected heterozygosity; *F*_IS_, Wright’s Fixation Index) according to changes in percent of missing data for the only known population of *M. catharinensis*.

% MD	SNPs	HWE^1^	LD^2^	Blast^3^	Filtered SNPs	*H_O_*	95% CI	*uH_E_*	95% CI	*F*_IS_	95% CI
0	139	(10); *p<* 3.6×10^−5^	(00); *k* =9.59×10^4^, *p* <5.2×10^−7^	01 (cp)	128	0.245	0.183, 0.307	0.205	0.160, 0.250	−0.145	−0.189, −0.101
5	283	(19); *p<* 1.8×10^−5^	(00); *k* =3.99×10^5^, *p* <1.2×10−^7^	01 (cp)	263	0.269	0.201, 0.337	0.224	0.174, 0.274	−0.147	−0.207, −0.087
10	591	(50); *p* <8.5×10^−6^	(00); *k* =1.74×10^6^, *p* <2.9×10^−8^	01 (cp)	540	0.283	0.210, 0.356	0.233	0.181, 0.285	−0.152	−0.221, −0.083
15	733	(65); *p* <6.8×10^−6^	(00); *k* =2.68×10^6^, *p* <1.9×10^−8^	01 (cp)	667	0.287	0.213, 0.361	0.234	0.185, 0.291	−0.148	−0.221, −0.075
20	1,019	(97); *p* <4.9×10^−6^	(00); *k* =5.18×10^6^, *p* <9.6×10^−9^	01 (cp)	921	0.293	0.217, 0.369	0.246	0.192, 0.300	−0.138	−0.223, −0.053
25	1,404	(118); *p* <3.6×10^−6^	(00); *k* =9.84×10^6^, *p* <5.1×10^−9^	01 (cp)	1,285	0.277	0.199, 0.355	0.238	0.181, 0.295	−0.115	−0.206, −0.024
30	1,634	(136); *p* <3.1×10^−6^	(00); *k* =1.33×10^7^, *p* <3.74×10^−9^	01 (cp)	1,497	0.268	0.188, 0.348	0.233	0.174, 0.292	−0.105	−0.199, −0.011

1 Number of SNPs with significant departures from Hardy–Weinberg equilibrium (HWE) after a Bonferroni adjustment.

2 Number of SNPs with significant departures from Linkage Disequilibrium (LD) after a Bonferroni adjustment.

3 Number of SNPs that matched some regions of the chloroplast (cp) and/or mitochondrial (mt) reference genomes.

### Genetic Diversity Parameters, Inbreeding Coefficient, and Outcrossing Rate

Genetic diversity parameters (*uH_E_* and *H_O_*) and Wright’s Fixation Index (*F*_IS_) for data sets with different percentages of missing data are shown in Table 1. For all genetic indices (*uH_E_*, *H_O_*, and *F*_IS_) obtained for the *M. catharinensis* population, no statistical differences were observed among the data sets (Table 1). Further analyses were performed using the data set without missing data, which presented a minor allele frequency (MAF) averaged at 0.125 (±0.093 SD).

Based on mating system analysis for the data sets with different percentages of missing data (Table 1), the outcrossing rates varied from 0.883 (SE±0.0483) to 0.909 (SE±0.011). Although the outcrossing rate was inflated with an increase in MD, no statistical differences were observed among the data sets (Supplementary Table S1).

### Clonality Assessment

**Figure 3:**
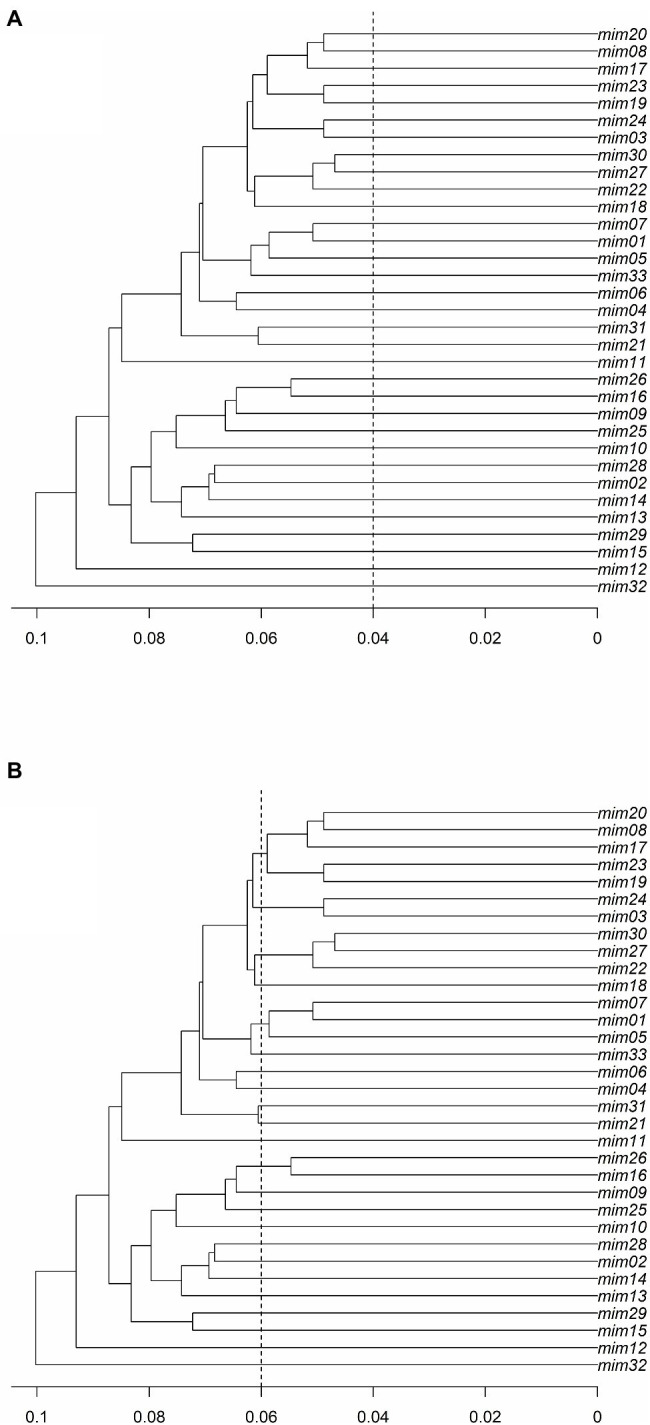
The ultrametric topology trees for the *M. catharinensis* population based on genetic distances (horizontal axis) and using a threshold of **(A)** 0.04 and **(B)** 0.06.

The results of the *PD* analysis showed that at least 13 loci are enough to discriminate closely related individuals with high accuracy, indicating that the data set with 0% missing data (i.e., 128 SNPs) has high discriminatory power to identify *M. catharinensis* individuals. The defined threshold of 0.04 did not cluster MLGs into multilocus lineages (MLLs; Figure 3A). While the genetic distance of 0.04 did not indicate the presence of clonal genotypes for *M. catharinensis*, the threshold of 0.06 indicates 23 putative MLLs (Figure 3B).

### Demographic History

The highest likelihoods are summarized in Table 2, as well as the results for the Akaike estimates (ΔAIC and *w*_i_). The model with the best fit for the complete (*N*=33) and the reduced (*n*=23) data sets was the bottleneck (Table 2). The inferred parameters for the bottleneck model and the 95% CIs obtained for the data sets with different numbers of individuals are shown in Supplementary Table S2. The parameter estimates under the empirical SFS suggests that population contraction occurred only a few generations ago (TBOT_compl_=82; TBOT_33_=10), with a continuous decline in population size after the bottleneck. However, the 95% CIs were wide and overlapping.

**Table 2:** Comparison among demographic models for the *M. catharinensis* population.

I^1^/Model	*k* ^2^	Max Est Lhood^3^	Max Obs Lhood^4^	AIC^5^	ΔAIC^6^	*w_i_* ^7^
33						
Neutral	1	−179.30	−148.16	823.72	9.50	8.0×10^−4^
**Bottleneck**	7	−175.06	−148.16	814.21	0.00	1.0×10^0^
Expansion	4	−179.21	−148.16	833.31	19.09	7.1×10^−6^
Decline	4	−179.23	−148.16	833.38	19.17	6.8×10^−6^
23						
Neutral	1	−579.40	−143.06	1160.81	754.98	1.1×10^−165^
**Bottleneck**	7	−195.91	−143.06	405.82	0.00	1.0×10^0^
Expansion	4	−518.76	−143.06	1045.53	639.70	1.2×10^−140^
Decline	4	−296.43	−143.06	600.876	195.05	4.4×10^−44^

The most plausible demographic scenario for each dataset is in bold.

1 Number of individuals used in the SFS (Site Frequency Spectrum) estimates.

2 Number of independently adjusted parameters within the model.

3 Maximum estimated likelihood.

4 Maximum observed likelihood.

5 Akaike’s information criterion (2*k*-2lnLhood).

6 Difference between the calculated AIC and the minimum AIC (AIC_i_–AIC_min_).

7 Akaike’s weight.

## Discussion

Genomic approaches are valuable as they can provide new insights and a better understanding of the amount, distribution, and functional significance of genetic variation in natural populations (Allendorf et al., 2010). As predicted by Funk et al. (2012), the use of genomic data is on the rise for species in relation to discussions around conservation (Hawkins et al., 2018; Huy et al., 2018; Lanes et al., 2018; Ball et al., 2020; Bao et al., 2020; Chen et al., 2020; Liu et al., 2020; Zang et al., 2020; Zimmerman et al., 2020; Bradbury et al., 2021). Thus, such data can play a critical role in informing management strategies and policy for species on the verge of extinction, many of which are narrow endemics and prime targets for conservation genomic assessments (Silva et al., 2020). Considering that estimates of genetic parameters have a direct influence on conservation decision-making, we applied a reduced representation library approach (i.e., ddRADseq) to generate genomic data for *M. catharinensis*. This data was used to assess intrapopulation genetic variation, as well as the demographic history for this critically endangered, narrow endemic species of the Atlantic Rainforest.

Our findings show that, contrary to what is expected for narrow endemics (Kimura and Crow, 1964; Hamrick and Godt, 1989; Spielman et al., 2004; Honnay and Jacquemyn, 2007), and despite its extremely small population size, *M. catharinensis* has moderate intrapopulation genetic diversity, expressed here by *H*_E_, with an apparent absence of inbreeding. Although the use of only one metric to assess genetic diversity is not an optimal condition, our results bring to light several factors that can influence the observed patterns of genetic diversity and inbreeding in relictual populations of threatened species. Our results underscore the importance of assessing the genetic patterns of each species in order to critically evaluate their genetic variability and the microevolutionary forces by which they are shaped (Amos and Harwood, 1998; Turchetto et al., 2016). Below, we discuss how our findings can inform conservation and management strategies for *M. catharinensis*.

### Contradicting Patterns of Genetic Diversity in Small Populations

Despite the general expectation of low intrapopulation genetic variability and high susceptibility to inbreeding depression associated with pronounced effects of genetic drift in small populations (e.g., Kimura and Crow, 1964; Hamrick and Godt, 1989; Barret and Kohn, 1991; Ellstrand and Elam, 1993; Honnay and Jacquemyn, 2007), the results for *M. catharinensis* do not support the hypothesized loss of genetic diversity, which should reflect high levels of inbreeding, as a consequence of reduced population size and narrow endemism. Although contradictory to theoretical predictions, unexpectedly high levels of genetic diversity are not unusual for narrow endemics (e.g., Fernández-Mazuecos et al., 2014; Jiménez-Mejías et al., 2015; Turchetto et al., 2016; Forrest et al., 2017; Goetze et al., 2018; Sękiewicz et al., 2020; Bard et al., 2021; Garcia-Jacas et al., 2021), creating an indistinct pattern for this kind of plant species. For instance, in the studies by Fernández-Mazuecos et al. (2014) and Jiménez-Mejías et al. (2015), the authors highlight the paradox of genetic diversity levels in narrow and extremely narrow endemic plant species from the Mediterranean. While Fernández-Mazuecos et al. (2014) found moderate levels of genetic diversity for *Naufraga balearica*, Jiménez-Mejías et al. (2015) reported high levels of genetic diversity for *Pseudomisopates rivas-martinezii*. In comparison to previous genetic assessments for narrow endemics in the same regions, the authors (Fernández-Mazuecos et al., 2014; Jiménez-Mejías et al., 2015) found that, although some species have low genetic variability, most species showed moderate to high levels of genetic diversity (e.g., Sales et al., 2001; Coppi et al., 2008; Mameli et al., 2008; Mayol et al., 2012; De Castro et al., 2013; Forrest et al., 2017; Sękiewicz et al., 2020; Garcia-Jacas et al., 2021). Thus narrow endemism does not always imply limited genetic diversity. However, such contrasting results may be due to differences in population size, distribution range, ecological traits, and evolutionary history (Fernández-Mazuecos et al., 2014).

In addition, several studies have indicated that life-history traits (e.g., lifecycle, growth form, mating and breeding systems) strongly influence the amount and distribution of genetic variation in natural populations (e.g., Hamrick and Godt, 1996; Ellis et al., 2006; Elegren and Galtier, 2016; Goetze et al., 2018; Bard et al., 2021; De Kort et al., 2021). For *Aechmea kertesziae*, a narrow endemic species of Brazilian Coastal *restingas* (sandy vegetation), traits such as self-incompatibility, long-term persistence, clonal reproduction, and consistent population size may explain the high levels of genetic diversity observed for the species (Goetze et al., 2018). Specifically for *M. catharinensis*, the predominance of historical outcrossing, as shown in our analysis, seems to be an important factor that contributes to the moderate levels of intrapopulation genetic diversity. It is important to note that outcrossing plants are likely to present higher levels of intrapopulation genetic diversity than self-compatible species (Hamrick and Godt, 1996; Glémin et al., 2006; Ball et al., 2020). Moreover, a lack of successful sexual reproduction in *M. catharinensis* (i.e., production of pods without seeds) suggests that the level of intrapopulation genetic diversity has remained static for at least two decades, even though our demographic inferences indicated a recent decline in population size.

As life-history traits play a significant role in shaping the patterns of genetic diversity, it is important to compare the levels of genetic diversity among congeneric species in order to mitigate phylogenetic effects (Bevill and Louda, 1999; Simon and Hay, 2003). This is essential to better understand how features such as population sizes and geographic distribution ranges affect reproductive biology and, as a consequence, the genetic variability of closely related plant species. For example, population genetics studies indicate that *Petunia secreta* and *Petunia exserta*, both of which are narrow endemic species, have high genetic diversity indices; however, these indices are lower than those reported for its congener *Petunia axillaris*, which has a wider geographic distribution range (Turchetto et al., 2016). Beyond dissimilarities in the area of occurrence of these *Petunia* species, different floral syndromes and reproductive systems have been described as the probable causes of the observed genetic variation (Kokubun et al., 2006; Turchetto et al., 2016).

Although *Mimosa* is one of the largest genera from Mimosoideae (Barneby, 1991), there are few population genetics studies for species of this genus (Moreira et al., 2011; Pramual et al., 2011; Arruda et al., 2019; Araújo et al., 2020; Morales et al., 2020), and none of these previous studies used SNP markers. Nevertheless, genetic diversity has been estimated using markers such as RAPD (*Mimosa pigra*; Pramual et al., 2011), AFLP (*Mimosa* subser. Dolentes–Brevipedes; Morales et al., 2020), ISSR (*M. caesalpiniifolia* Benth.; Araújo et al., 2020), and allozyme (*Mimosa scabrella*; Moreira et al., 2011; Arruda et al., 2019). In this context, *M. catharinensis* presented lower levels of intrapopulation genetic diversity than that reported for its widespread congener *M. scabrella* (*H_E_* =0.362 to 0.469; Moreira et al., 2011; Arruda et al., 2019).

When comparing studies on narrow endemics assessed using SNPs, and considering the theoretical maximum heterozygosity for biallelic markers (*H_E_* =0.5), we are unable to make generalizations about the genetic diversity of narrow endemics. Indeed, *M. catharinensis* displays higher levels of genetic diversity than self-compatible (e.g., Ball et al., 2020) and clonal and functional sterile species (e.g., Amor et al., 2020). However, some tropical plant species (e.g., Lanes et al., 2018; Ball et al., 2020) showed the most similar genetic diversity to *M. catharinensis*.

### Inbreeding and Mating System of *Mimosa catharinensis*

Our findings indicate an excess of heterozygosity for this plant species, counteracting the theoretical expectation that reductions in population size will result in high levels of inbreeding. In terms of genetic variability, the levels of inbreeding in narrow endemic plant species with small population sizes vary according to individual features of each species/population (Angeloni et al., 2011). Reports for plant species with very small populations vary from high levels of inbreeding (Jiménez et al., 2014; Finlay et al., 2017; Rodrigues et al., 2019; Wang, 2020), to no evidence of inbreeding (Edwards et al., 2014, 2021; Spoladore et al., 2017), to an excess of heterozygotes (Cabrera-Toledo et al., 2008; Radosavljević et al., 2015; Amor et al., 2020) as found herein. Barret and Kohn (1991) emphasize that the mating pattern is a prime determinant of inbreeding levels in natural populations regardless of their size. In fact, self-compatible species can be more susceptible to inbreeding considering that self-incompatibility is likely to have evolved to prevent inbreeding depression (Charlesworth and Charlesworth, 1987). On the other hand, predominantly outcrossing species can suffer from inbreeding depression due to mating between relatives (Husband and Schemske, 1996).

The (historical) outcrossing rate observed in *M. catharinensis* suggests that it has a mixed mating system, which is characterized as ranging from 5 to 95% depending on environmental conditions and pollination frequency (Karasawa, 2015). Such a wide range in outcrossing rates for mixed-mating species are common and can change over time and space due to environmental conditions and intrinsic population features, such density and demographic structure (e.g., Wright et al., 2013). As with *M. catharinensis*, high outcrossing rates have been reported for many Neotropical plant species (e.g., Ward et al., 2005; Feres et al., 2012, 2021; Nazareno and Reis, 2012; Spoladore et al., 2017; Godoy et al., 2018; Montagna et al., 2018; Sujii et al., 2021), including two congener *Mimosa* species (Moreira et al., 2011; Arruda et al., 2020). Studies on populations of *M. scabrella* (Arruda et al., 2020), for instance, showed a similar reproductive pattern with high outcrossing rates (*t*=0.925/0.845) and low estimates of selfing (*s*=0.075/0.155). For *M. catharinensis*, the interpretation of negative *F*_IS_ values coupled with high *t* values should be viewed with caution due to the distinct biology of the species (i.e., production of seedless pods and absence of seedlings in the study area). Such biological characteristics observed for *M. catharinensis* suggest that a significant selective pressure against homozygotes must have occurred in previous generations, especially when taking into account the excesses of heterozygosity observed in its small population. As *M. catharinensis* population is constituted only by adult trees, a further study investigating inbreeding depression seems not to be a feasible task as a consequence of the lack of different ontogenic stages in its population.

Although pollen grains have been reported as viable for *M. catharinensis* (Silva et al., 2005), the apparent reproductive failure seems to be due to an extremely low frequency of floral visitors (Silva et al., 2005), implying low pollination efficiency. This phenomenon has been also described for other *Mimosa* species, such as *Mimosa bimucronata* (Seijo and Neffa, 2004). As with *M. catharinensis*, *M. bimucronata* does not produce nectar, however, its newly opened flowers exude a slight fruit odor to attract pollinators (Silva et al., 2011). Despite poor fruit set due to inefficient pollination, *M. bimucronata* produces viable fruits and seeds (Seijo and Neffa, 2004). In contrast, *Mimosa pudica* showed high pollination efficiency, mediated by *A. mellifera*, resulting in high fruit and seed yields and quality (Taimanga and Fohouo, 2018). Further pollination biology studies are needed to clarify the causes of reproductive failure in *M. catharinensis*.

### Reconstruction of the Demographic History of *Mimosa catharinensis*

The selection of the best demographic model was the main focus of the coalescent-based inference for *M. catharinensis*, considering a comparison of two data sets with different numbers of individuals and excluding putative clonal individuals based on their genetic distance. The fact that confidence intervals were wide and overlapping did not affect our results and can be related to the uncertainty of the estimated parameters and/or the small SFS (Rödin-Mörch et al., 2019). Nonetheless, the values presented in Supplementary Table S2 should be interpreted with caution. The generated SFS for both data sets suggest that a recent bottleneck is the best explanation for the current levels of genetic diversity in *M. catharinensis*. This corroborates our findings of excess heterozygosity for the species, which is a characteristic of populations that have experienced a recent bottleneck (Luikart et al., 1998). Of the factors that may have resulted in a drastic decrease in species population sizes in the *restinga*, the fragmentation of Santa Catarina Island’s native vegetation is particularly relevant, especially the degradation that occurred within the PAERVE before it was deemed a protected area. The implementation of reforestation projects in the park using exotic, invasive species also resulted in fires, changes to the soil, and deforestation of native vegetation, all of which accentuated the damage occurring to such a heterogeneous vegetal formation (Heberle, 2011).

In contrast to the evidence of population contraction for *M. catharinensis*, the majority of studies on past demographic changes in tropical plant species from South America have reported indications of expansion during the Quaternary period (e.g., Turchetto-Zolet et al., 2013). On the other hand, demographic stability has been suggested for the period for species from the Southern Brazil coastal *restinga* (Goetze et al., 2018). Therefore, further studies are needed to infer historical changes in population sizes of plant species exclusive to Santa Catarina Island (Hassemer et al., 2015), whose recent demographic changes may be similar to that reported herein for *M. catharinensis*.

### Implications for Conservation

The extremely small number of individuals (*N*=33) in the only known *M. catharinensis* population, coupled with strong evidence for a lack of sexual reproduction, suggests that the conservation perspectives for this critically endangered species are concerning. Although no inbreeding was detected, the species is facing imminent risk of extinction because of its reduced population size, making it more susceptible to stochastic events. As a matter of fact, *ex situ* conservation activities must be implemented in order to safeguard the remaining genetic diversity of *M. catharinensis*. One potential strategy suggested in studies on biodiversity conservation of threatened species consists of the preservation of tissue culture, an *in vitro* cultivation of isolated live tissue, enabling the propagation of species facing difficulties with natural reproduction (Paiva and Paiva, 2001; Santos et al., 2019). Protocols to apply tissue culture techniques for conservation of endangered species have been successfully developed (e.g., Sherif et al., 2018; Choudary et al., 2020; Lerin et al., 2021; Mishra et al., 2020), and such an approach has been used to conserve the narrow endemic species *Styphelia longissima* (Thomas et al., 2021).

In addition, as *in vitro* germination tests show pollen grain viability (Silva et al., 2005), additional pollination biology studies (e.g., pollination treatments such as manual cross-pollination and self-pollination) should be performed to test for viable seed production. The information available through pollination biology studies can help us to solve the puzzle of *M. catharinensis* reproduction, allowing us to better plan effective conservation measures for this rare plant species. In terms of *in situ* conservation, the fact that the species is found in a protected area is a significant starting point, although to date this fact has been insufficient for its conservation. Today, the management of invasive, exotic species (e.g., *Pinus* spp. and *Eucalyptus* spp.), one of the main issues threatening the flora of PAERVE, should be prioritized. Furthermore, it is widely reported that adequate restoration of the contaminated areas of the park are urgently needed (Ferreira, 2010; Bechara et al., 2013), and efforts to achieve this goal have been implemented according to the park’s Management Plan (IMA, 2020). Moreover, although a robust floristic survey of Santa Catarina State has been conducted (Vibrans et al., 2012), not all the biodiversity present in the state was sampled. As such, biodiversity inventories within Brazil’s protected areas are crucial (Oliveira et al., 2017), and efforts on field surveys must be undertaken to verify the narrow occurrence of this critically endangered plant species. Likewise, we are committed to monitoring the *M. catharinensis* population size over time to avoid further losses in genetic diversity.

## Data Availability Statement

SNP data sets for M. catharinensis are available for download from the Dryad Digital Repository (https://doi.org/10.5061/dryad.j9kd51ccz).

## Author Contributions

AN designed the study, collected the samples, and conducted molecular work. TT performed analyses, and led the writing of the manuscript with input from AN, who also provided analytical support. All authors contributed to the article and approved the submitted version.

## Funding

We thank the Conselho Nacional de Desenvolvimento Científico e Tecnológico (CNPq) to AN (429266/2018-9) for funding the field and molecular work associated with this project. Additional funds were provided by the Conselho Nacional de Desenvolvimento Científico e Tecnológico (CNPq) through a PQ-2 grant to AN (306182/2020-3) and the Fundação de Amparo à Pesquisa de Minas Gerais (FAPEMIG) for the scholarship to TT.

## Conflict of Interest

The authors declare that the research was conducted in the absence of any commercial or financial relationships that could be construed as a potential conflict of interest.

## Publisher’s Note

All claims expressed in this article are solely those of the authors and do not necessarily represent those of their affiliated organizations, or those of the publisher, the editors and the reviewers. Any product that may be evaluated in this article, or claim that may be made by its manufacturer, is not guaranteed or endorsed by the publisher.

## Footnotes

^1^ http://www.bioinformatics.babraham.ac.uk/projects/fastqc/

^2^ https://CRAN.R-project.org/package=adegenet

^3^ http://blast.ncbi.nlm.nih.gov

^4^ https://CRAN.R-project.org/package=diveRsity
