# Supplementary material for: One Step Away From Extinction: A Population Genomic Analysis of A Narrow Endemic, Tropical Plant Species
Source: Front Plant Sci. 2021 Sep 23;12:730258. doi: 10.3389/fpls.2021.730258 (PMC8496504; doi:10.3389/fpls.2021.730258)
Supplement: Supplementary file 1 [file Table_1.DOCX]

**ONE STEP AWAY FROM EXTINCTION: A POPULATION GENOMIC ANALYSIS OF A NARROW ENDEMIC, TROPICAL PLANT SPECIES**

**Supporting Information**

THAIS M. TEIXEIRA1, ALISON G. NAZARENO1,2*

^1^ Department of Genetics, Ecology and Evolution, Federal University of Minas Gerais, Belo Horizonte, MG, Brazil.

^2^ Department of Ecology and Evolutionary Biology, University of Michigan, Ann Arbor, MI, USA.

**TABLE S1** Selfing (*s*) and outcrossing (1-*s*) rates estimated for *Mimosa catharinensis* according to changes in percent of missing data (MD).

| MD % | *s* | 1-*s* | 95% CI* |
| --- | --- | --- | --- |
| 0 | 0.116 | 0.883 | 0.789 - 0.977 |
| 5 | 0.115 | 0.885 | 0.820 - 0.949 |
| 10 | 0.100 | 0.900 | 0.862 - 0.937 |
| 15 | 0.094 | 0.906 | 0.872 - 0.939 |
| 20 | 0.102 | 0.898 | 0.870 - 0.925 |
| 25 | 0.098 | 0.902 | 0.879 - 0.925 |
| 30 | 0.091 | 0.909 | 0.887 - 0.930 |

*95% confidence intervals calculated for 1-*s.*

**TABLE S2** Inferred parameters for the bottleneck model for *Mimosa catharinensis* considering the complete (N=33) and the reduced (n=23) data sets. The 95% confidence intervals were obtained based on 100 parametric bootstraps.

| Parameter | Complete | 95%CI | Reduced | 95%CI |
| --- | --- | --- | --- | --- |
| NCUR^1^ | 2157.0 | 168.0 - 11082.5 | 530.0 | 13.0 - 18941.0 |
| NANC^2^ | 108210.0 | 49.0 - 105906.0 | 1045457.0 | 25.5 - 1045457.0 |
| NBOT^3^ | 13934.0 | 9198.5 - 97765.5 | 97872.0 | 10124.0 - 104697.5 |
| TBOT^4^ | 82.0 | 16.0 - 8106.0 | 10.0 | 13.5 - 9113.5 |

^1^ Current population size.

^2^ Ancestral population size.

^3^ Population size at the end of the bottleneck.

^4^ Number of generations since the bottleneck occurred.
